# Efficacy and safety of *Latilactobacillus curvatus* LB-P9 on hair health: a randomized, double-blind, placebo-controlled clinical trial

**DOI:** 10.3389/fnut.2024.1447863

**Published:** 2024-11-12

**Authors:** Sun Young Choi, Eun Jung Ko, Joon Seok, Hye Sung Han, Kwang Ho Yoo, MiKyung Song, Kyoungsub Song, Beom Joon Kim

**Affiliations:** ^1^Department of Dermatology, Chung-Ang University Gwangmyeong Hospital, Chung-Ang University College of Medicine, Gwangmyeong-si, Gyeonggi-do, Republic of Korea; ^2^Department of Dermatology, National Police Hospital, Seoul, Republic of Korea; ^3^Department of Dermatology, Chung-Ang University Hospital, Chung-Ang University College of Medicine, Seoul, Republic of Korea; ^4^R&D Center, LISCure Biosciences Inc., Seongnam, Republic of Korea

**Keywords:** hair health, probiotics, hair luster, hair elasticity, *Latilactobacillus curvatus* LB-P9

## Abstract

**Introduction:**

Numerous factors influence hair health, including genetic predisposition, hormonal changes, stress, nutritional deficiencies, medical conditions, or medications. With the rising interest in maintaining hair health, alternative approaches such as functional cosmetics and food products are gaining attention. Probiotics, health-beneficial live microorganisms, are emerging as potential candidates for improving hair health. Therefore, this study aimed to evaluate the effects and safety of oral intake of *Latilactobacillus curvatus* LB-P9 on hair health.

**Methods:**

The study was a randomized, double-blind, placebo-controlled clinical trial involving participants (aged 18–60 years old) with mild to moderate hair damage. Participants were randomly assigned to the test (receiving LB-P9 supplements) or control (receiving a placebo) groups, respectively. Efficacy was assessed using measures such as hair luster, elasticity, and participant satisfaction. Safety evaluations comprised physical examinations, vital sign measurements, laboratory tests, and observation of adverse reactions.

**Results:**

Overall, 80 participants were enrolled in the trial. Significant improvements were observed in hair luster, elasticity, and participant satisfaction in the test group compared to the control group. In the test group, the hair luster parameter increased by 1.65 ± 2.30 (L_BNT_) at 24 weeks (*p* < 0.001), indicating a 19% improvement over the control group. Subgroup analysis revealed significant improvement in hair luster among females with short hair. Additionally, hair tensile strength, reflecting hair elasticity and participant satisfaction are increased by 10.27 ± 16.40 (gf/mm^2^) at 24 weeks (*p* = 0.001) in the test group. The subjective indicator of participant satisfaction, which improves as survey scores decrease, significantly decreased in the test group by −17.81 ± 14.35 points (*p* < 0.001) after 24 weeks of consuming the test food than before consuming it. No significant adverse reactions were reported, and safety evaluations indicated no adverse effects linked to LB-P9 consumption.

**Conclusion:**

Probiotics, including LB-P9, may serve as an alternative in the management of hair health. The findings of this study support the possible benefits of LB-P9 supplementation in enhancing hair luster and elasticity.

## Introduction

Hair health is a multifaceted concept that encompasses various physical and biochemical properties of the hair shaft and scalp. Healthy hair is not only a key indicator of overall well-being but also a critical factor in an individual’s self-esteem and social interactions. The concept of hair health extends beyond esthetic appeal, as it reflects the condition of the scalp, the integrity of the hair fibers, and the balance of moisture, proteins, and lipids within the hair (1–3).

Several indicators are commonly used to assess hair health, each reflecting different aspects of hair quality (4–8). Hair luster is one of the most visually apparent indicators of hair health. It refers to the light-reflecting properties of the hair surface, which are influenced by the smoothness of the cuticle layer and the alignment of hair fibers. High luster is associated with well-conditioned hair that has a smooth cuticle, which allows light to reflect evenly. On the contrary, dull hair often indicates damage, dryness, or poor cuticle condition (4, 5). Other factors include hair elasticity, porosity and scalp condition. Hair elasticity is the ability of hair to stretch and return to its original shape without breaking. It is largely dependent on the protein structure of the hair shaft, particularly keratin. Hair porosity refers to the ability to absorb and retain moisture of hair. This characteristic is determined by the condition of the hair cuticle (9, 10). Healthy hair exhibits high luster, good elasticity and porosity reflecting a scalp that is free from dandruff, inflammation, and excessive sebum production. Each of these indicators provides insights into the structural and biochemical integrity of the hair. Understanding these parameters is essential for developing targeted treatments and maintenance routines aimed at preserving or restoring hair health (6–8).

In recent years, interest in functional cosmetics and food products for hair loss has increased, reflecting a growing need for basic hair health improvement. These approaches offer benefits in terms of affordability and safety, leading to an increase in demand. Consequently, various natural and herbal products are increasingly utilized for hair loss and health in local and international markets. Researchers are exploring alternative treatments, particularly the potential therapeutic efficacy of natural products, focusing on their underlying mechanisms of action and identifying their actual side effects. Continuous studies are necessary to determine the effectiveness of these treatments as viable therapeutic options (11–13).

Probiotics are being proposed as alternative treatment strategies for promoting healthy hair (14, 15). These live microorganisms are known to provide health benefits to the host, and their use for maintaining overall health and well-being is increasing globally. Despite conflicting clinical outcomes for various probiotic strains and formulations, their popularity continues to grow (16, 17). Many studies are currently exploring whether specific probiotics can induce changes in human physiological parameters, with a specific focus on tissues or organs (18–21). This association is often linked to the gut-specific organ axis. Recent research on the gut-brain-skin axis suggests that probiotics may influence skin inflammation, hair cycling, and growth (18–21). Therefore, this study aims to evaluate the effects and safety of orally consumed *Latilactobacillus curvatus* LB-P9—a functional ingredient developed for use in healthy functional foods—regarding its impact on hair health. The strain used in this clinical study was selected through a screening process in our preclinical research, which identified strains that increase the expression of hair growth-inducing genes such as vascular endothelial growth factor (VEGF), fibroblast growth factor 7 (FGF-7), and insulin-like growth factor-1 (IGF-1).

## Methods

### Study design

This study was designed as a randomized, double-blind, placebo-controlled clinical trial. Participants were aged 18–60 years old with mild to moderate hair damage and no underlying scalp conditions or hair loss disease. The inclusion criteria for hair luster were specifically clarified as follows: participants had hair luster scores of 1, 2, or 3 points, based on a five-grade visual assessment classification method (1 point for lusterless, 2 points for somewhat lusterless, 3 points for intermediate, 4 points for somewhat lustrous, and 5 points for lustrous) (7). The degree of hair damage was assessed through a separate total hair damage scare based on exposure to risk factors. After checking the frequency of hair damage behaviors and summing up the corresponding scores, the degree of hair damage was assessed as mild (less than 7 points), moderate (7 or more, but less than 18 points), and severe (18 points or more) (Supplementary Table 1) (8).

The inclusion criteria were individuals who did not use any special hair products or treatments during the study period, agreed to maintain the same hairstyle and length, and had hair length of at least 8 cm long measured from the top of the scalp. The exclusion criteria included underlying scalp conditions or hair loss disease, acute renal or cardiovascular disease within the past 6 months, uncontrolled hypertension, diabetes, hyperthyroidism or hypothyroidism, chronic kidney failure, and severe gastrointestinal symptoms, including lactose intolerance. Individuals were excluded if their serum creatinine, AST, or ALT levels exceeded twice the upper limit of normal for the research institution at screening. The exclusion criteria also included individuals who were administered oral dutasteride or finasteride in the past 6 months, applied topical hair loss treatments or growth stimulants within the past month, or consumed functional health supplements related to hair health improvement within the past 3 months.

Those who agreed to participate in the study by providing written informed consent, meeting the inclusion criteria, and did not meet the exclusion criteria were randomly assigned to the test or control group in a 1:1 ratio. Randomly assigned participants received test or control supplements, and their daily consumption was recorded according to the specified method. Concurrent use of health supplements or medications that were deemed not to affect the study was permitted during the study period. During the study period, participants were instructed to refrain from using hair products recognized by the Ministry of Food and Drug Safety for alleviating hair loss symptoms such as shampoo, conditioner, and scalp essence. Participants were instructed to only use shampoo and conditioner, limiting hair washing to no more than twice a day. Additionally, participants were advised not to undergo any hair treatments that provide nourishment to the hair, such as hair dyeing, bleaching, perming, or hairstyling using a flat iron during the study period.

The study protocol was approved by the Institutional Review Board of P&K Skin Research Center under reception number P2211-3738, and written informed consent was obtained from all participants after a full explanation of the risks and benefits of the procedure. This trial was registered in the International Clinical Trials Registry Platform of the WHO with the following identification number: KCT0009499.

### Supplements

The probiotics used in this study were *L. curvatus* LB-P9, originally isolated from Kimchi. The investigational product contained LB-P9 (60 mg) as the main ingredient and microcrystalline cellulose, lactose, magnesium stearate and silicon dioxide at 400 mg/capsule as excipients. The placebo drug contained microcrystalline cellulose, lactose, magnesium stearate and silicon dioxide as excipients at 400 mg/capsule. The daily intake of LB-P9 in the test group was 5 billion CFU/capsule/day as target dose. The clinical study was conducted with instructions to store the product in a refrigerator. The test and placebo capsules were manufactured to be similar in shape, size, and color. Identical labels were attached to the control and test supplements to ensure double blinding for the participants and the investigators.

All participants were instructed to take the test or control supplement once daily, in the evening after a meal, with water, for 24 weeks. It was designed to enhance participant compliance during the human application trial by scheduling the intake during the evening. The compliance rate was calculated as the percentage of the number of times the participants actually consumed divided by the number of times they were supposed to consume during the study period. Investigators determined the compliance rate by referencing the remaining supplements after consumption and reviewing the consumption diary recorded by the participants. Participants were considered compliant if they consumed ≥80% of the total prescribed dosage of the test product. Those who consumed the supplements inconsistently (with a compliance rate of <80%) or missed consumption for >5 consecutive days were excluded from the study.

### Target number of participants

The study hypothesizes that the use of LB-P9 will improve hair health. Therefore, a primary comparison will be conducted between the control group and the test group (LB-P9). Since there are no existing human trials for the test substance, we used data from studies on shampoo and hair tonic, which primarily contain natural extracts, to estimate the number of subjects (22). In the study using natural extracts shampoo and hair tonic over 6 weeks, hair luster increased from 4.04 to 5.24 in the test group, with no control group established. It is expected that the intake of the test supplements, LB-P9 will show similar results, estimating an improvement of 1.20 compared to the control group. The standard deviation of 1.67 after product use, as reported in the reference literature, was adopted as the common standard deviation. The significance level was set at 0.05 (two-sided) and the power at 80%. As a result of calculating the number of participants using G*Power (ver. 3.1.9.4), it was calculated to be 32 per group and a total of 64. After considering the dropout rate of 20%, the number of participants was calculated to be 40 per group and a total of 80.

### Efficacy evaluation

Efficacy assessment was conducted at 12 and 24 weeks after oral supplement intake. All efficacy evaluations were conducted by blinded investigators or blinded participants. The primary efficacy endpoint was the change in hair luster parameter (L_BNT_) measured at week 24 using a luster measurement device called SAMBA (Bossa Nova Technologies, USA). SAMBA utilizes a dual-polarized imaging system to quantify parameters such as luster, color, and hair image. Its components include a polarization color camera, polarized illumination, and a cylindrical mount. It applies the principle of measuring the physical characteristics of hair samples by simultaneously measuring the angle of light reflected by each hair fiber when light is projected onto the hair. When sampling hair for luster measurement using the SAMBA device, we maintained the indoor temperature at 22 ± 2°C and humidity at 50 ± 5%. After approximately 2 h of acclimation under these conditions, a hair bundle was selected, and imaging was conducted from three randomly selected areas, obtaining the average value.

The secondary efficacy evaluation variables included changes in hair elasticity (gf/mm^2^) and subjective satisfaction scores of participants for hair health, assessed using a questionnaire after 24 weeks. For the evaluation of changes in hair elasticity, tensile strength was measured. This involved pulling both ends of the hair at a constant pressure and a speed of 2 cm/min until the hair broke. Three randomly selected areas of the scalp were evaluated for tensile strength using a Universal Testing Machine (Model 4,301, Instron, USA). The average value from at least 10 measurements per area was used. To ensure accurate comparisons of hair breakage, hair samples were consistently taken from the same location in each measurement area for assessment. The questionnaire utilized for subjective satisfaction scores in this study is provided in Supplementary Table 2. Subjective satisfaction scores ranged from 8 points (minimum) to 80 points (maximum), with lower scores indicating a higher level of subjective satisfaction with hair health.

### Safety evaluation

At every visit (baseline and after 12 and 24 weeks of oral supplements), safety evaluations were performed. Participants underwent physical examinations and vital sign measurements. Blood tests, including complete blood count, liver, and kidney function tests, serum glucose levels, and serum inflammatory markers, were also conducted.

Investigators observed the hair and scalp of participants for objective signs of irritation, including erythema, edema, and papules or pustules. Subjective sensations reported by participants, including itching, stinging, burning, tingling, and roughness, were evaluated through interviews. Any adverse reactions that occurred during the study period were recorded.

### Statistical analysis

Statistical analyses were conducted using SPSS, Version 26.0 (IBM Corporation, Armonk, NY, USA). All statistical significance tests were performed at a significance level of 5%. The normality of continuous variables was assessed using the Shapiro–Wilk test. We analyzed between-group comparisons using the Student’s t-test and Wilcoxon’s rank sum test. We analyzed changes over time using mixed models. We conducted within-group comparisons using paired *t*-tests and Wilcoxon’s signed rank test. We conducted between-group comparisons using Wilcoxon’s rank sum test for ordinal variables and within-group comparisons using Wilcoxon’s signed rank test. If between-group differences in baseline hair damage scores could affect the evaluation of efficacy endpoints, additional analysis using covariance analysis was performed, considering these differences as a covariate. Subgroup analyses related to sex, hair length, and degree of hair damage were performed separately.

## Results

### Demographics

Eighty participants were initially enrolled in the clinical trial. After excluding seven dropouts, 36 and 37 participants in the test and control groups completed the trial, respectively. Among the seven dropouts, three withdrew their consent, two did not meet compliance criteria for product consumption, and two violated the selection/exclusion criteria. No statistically significant differences were observed between the baseline characteristics of participants in the test and control groups, indicating homogeneity between the two groups before supplement consumption (Table 1).

**Table 1:** Baseline characteristics of participants.

Variable		PP population		
		Control group (*n* = 37)	Test group (*n* = 36)	*p* value
Age (year)		41.35 ± 10.35	40.03 ± 11.97	0.615^1^
Height (cm)		161.53 ± 7.30	161.41 ± 6.82	0.728^2^
Weight (kg)		61.35 ± 12.72	61.77 ± 12.08	0.703^2^
Sex (*n*)	Male	7 (18.9%)	7 (19.4%)	0.955^3^
	Female	30 (81.1%)	29 (80.6%)	
Smoking	None	36 (97.3%)	32 (88.9%)	0.231^4^
	Past	0 (0.0%)	1 (2.8%)	
	Present	1 (2.7%)	3 (8.3%)	
Drinking	None	20 (54.1%)	21 (58.3%)	0.713^3^
	Moderate	17 (45.9%)	15 (41.7%)	
	Heavy	0 (0.0%)	0 (0.0%)	

Data were expressed mean ± standard deviation or frequency (proportion).

Shapiro–Wilk’s test was employed for test of normality assumption.

^1independent t-test.^

^2Mann–Whitney U test.^

^3Chi-square test.^

^4Fisher’s exact test.^

### Efficacy evaluation

**Figure 1:**
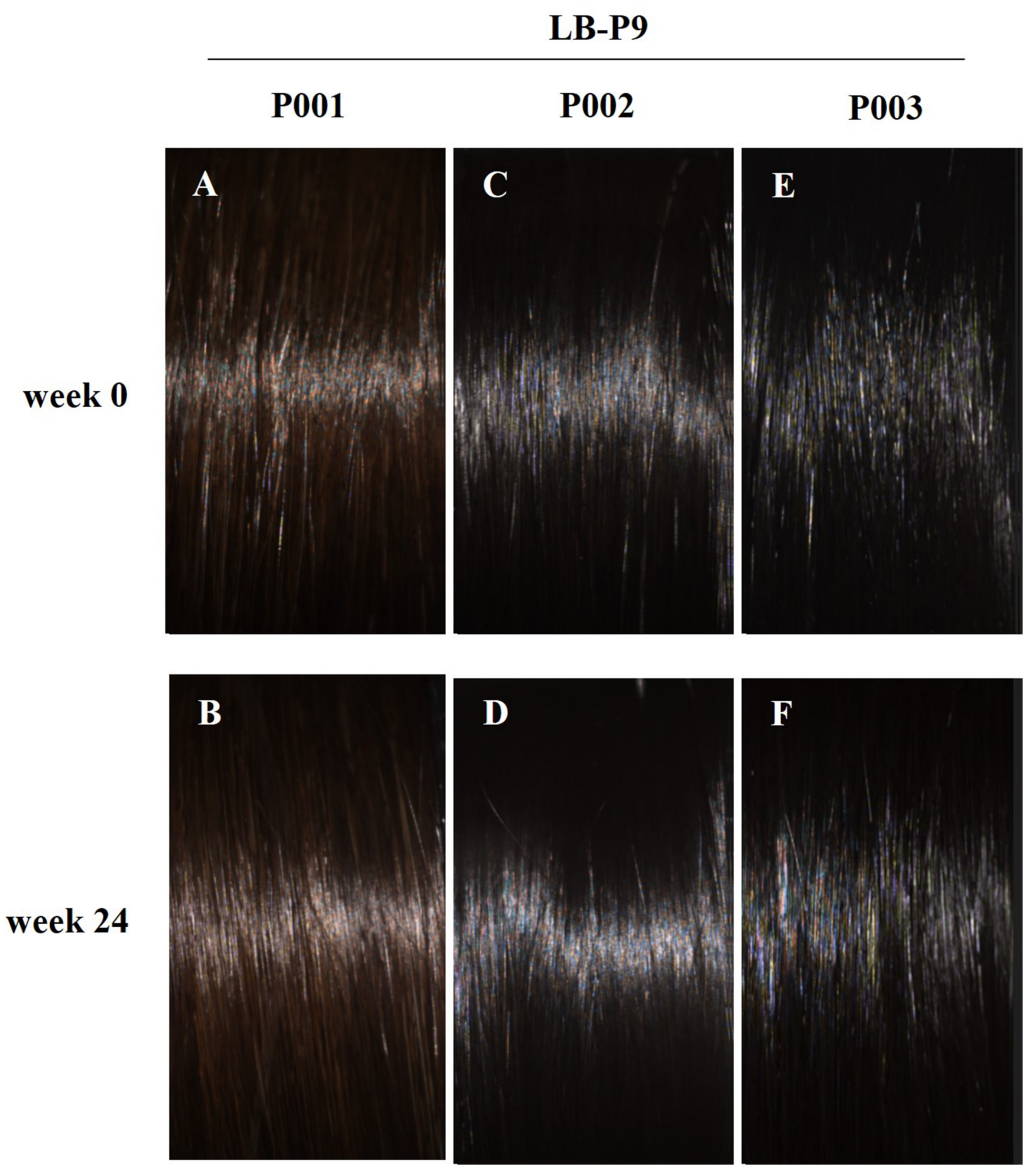
The images of hairs of representative participants (A,C,E: Treated of LB-P9 0-week; B,D,F: Treated of LB-P9 24-week).

The primary efficacy parameter, the change in hair luster parameter (L_BNT_) at 24 weeks compared to baseline, was 0.54 ± 1.87 and 1.65 ± 2.30 in the control and test groups, respectively. The results showed a statistically significant increase (*p* < 0.001) in hair luster within the test group when comparing pre-consumption and post-consumption of the test supplements. Additionally, a statistically significant increase (*p* = 0.027) was observed between the test and control group. Hair luster parameter at 24 weeks improved by approximately 19% in the test group compared to the control group. Figure 1 shows the representative images of participants from the test group showing improvement in hair luster.

**Figure 2:**
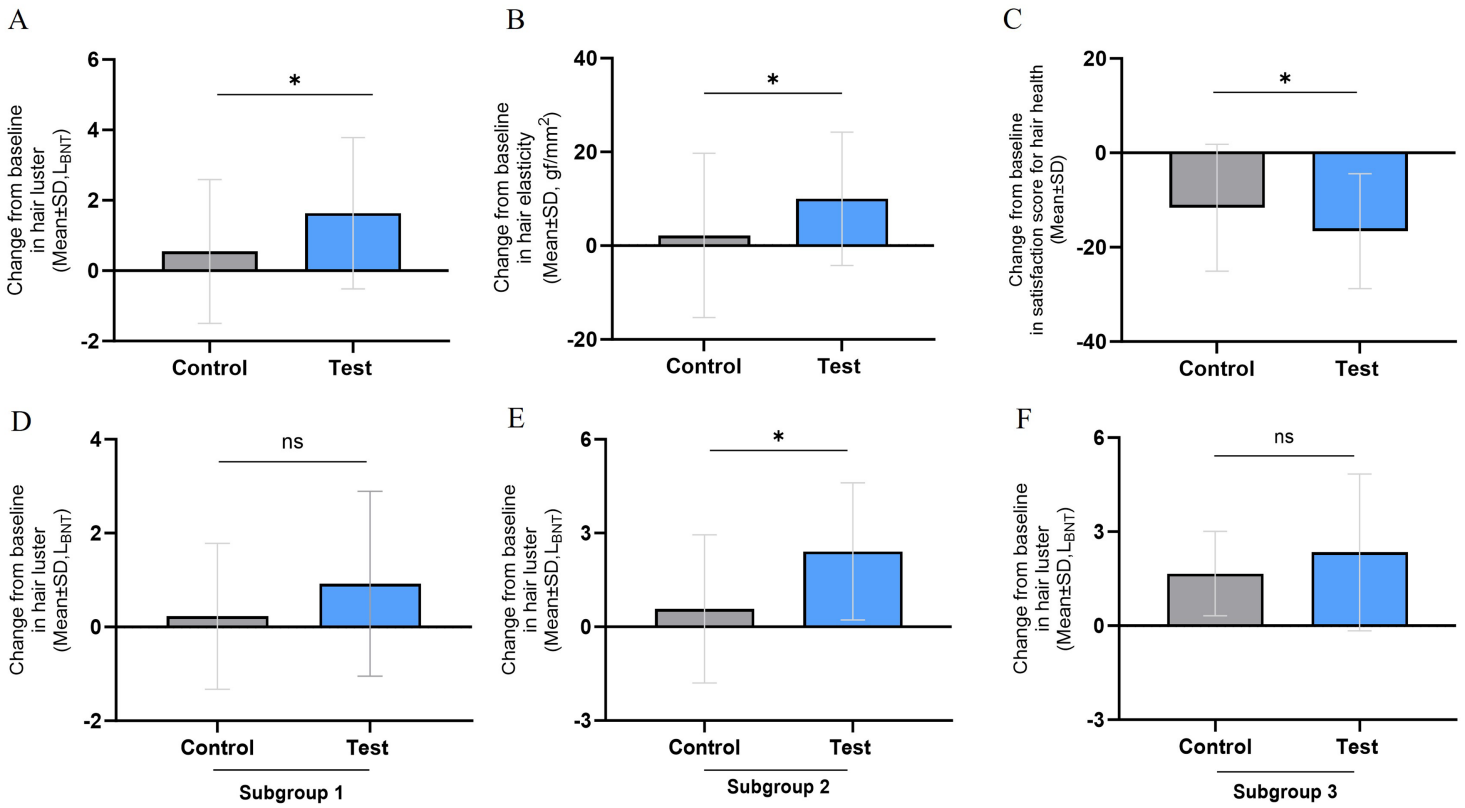
Evaluation of hair health effectiveness after 24 weeks (A ~ C) and subgroup analysis (D ~ F). (A) Change from baseline in hair luster, (B) Change from baseline in hair elasticity, (C) Change from baseline in satisfaction score for hair health. (D) Female with long hair of change from baseline in hair luster (*n* = 29), (E) Female with short hair of change from baseline in hair luster (*n* = 30), (F) Male with long and short hair of change from baseline in hair luster (*n* = 14). The results are presented as means ± SD, *p < 0.05.

Subgroup analysis based on sex and hair length was conducted to measure hair luster. The long hair is defined as being 8.0 cm or longer. In the subgroup analysis of females with long hair, the changes in hair luster was not significantly increased after 24 weeks compared to the baseline in the test group (*n* = 13 0.75 ± 1.56 L_BNT_, *p* = 0.106), and there was no significant difference change in hair luster compared to the control group (*n* = 16, 0.50 ± 2.02 L_BNT_, *p* = 0.483). Increasing changes in hair luster were observed after 24 weeks compared to the baseline in the test group, while no significant difference was found in the control group. Among females with short hair, a statistically significant increase in hair luster was observed after 24 weeks compared to baseline in the test group (*n* = 16, 2.07 ± 2.62 L_BNT_, *p* = 0.006), while no significant difference was observed in the control group (*n* = 14, 0.20 ± 1.72 L_BNT_, *p* = 0.673). Furthermore, compared to the control group, the increase in hair luster in the test group at 24 weeks remained statistically significant (*p* = 0.031). Conversely, in males, the change in hair luster after 24 weeks compared to baseline was statistically significantly increased in the test group (*n* = 7, 2.34 ± 2.50 L_BNT_, *p* = 0.048). However, when compared to the control group (*n* = 7, 1.31 ± 1.84 L_BNT_, *p* = 0.110), this increase was not statistically significant (*p* = 0.398). Figure 2 shows the details of the subgroup analysis.

Hair tensile strength—an indicator of hair elasticity—increased significantly in the test group by 10.27 ± 16.40 gf/mm^2^ (*p* = 0.001) after 24 weeks of consumption than before consumption, while the control group increased by 1.95 ± 15.45 gf/mm^2^ (*p* = 0.448). A statistically significant increase was observed in the test group than in the control group (Figure 2B). When comparing the changes in hair tensile strength between groups, the test group showed a statistically significant increase compared to the control group (*p* = 0.029).

The subjective indicator of participant satisfaction, which improves as survey scores decrease, significantly decreased in the test group by −17.81 ± 14.35 points (*p* < 0.001) after 24 weeks of consuming the test food than before consuming it. Compared within the groups, the control group experienced a reduction of −10.45 ± 10.49 points (*p* < 0.001), indicating a statistically significant reduction in both groups (Figure 2C). When comparing the change in satisfaction scores between the groups, the test group showed a statistically significant reduction compared to the control group (*p* = 0.015). Table 2 details measurements of the efficacy evaluation parameters. In the subgroup analysis, specifically in the group classified with hair damage scores from between 7 and 18 (inclusive), the change in participant satisfaction in the test group was −18.91 ± 14.38 points (*p* < 0.001), and the change in hair elasticity in the test group was 10.55 ± 16.94 gf/mm^2^ (*p* = 0.041). These results were found to be significant when compared between the groups Therefore, the subgroup analysis of change in participant satisfaction according to hair damage scores after 24 weeks indicates that differences between groups with *p* values less than 0.05 are statistically significant (Supplementary Figure 1).

**Table 2:** Efficacy assessment for hair health (Per Protocol, PP).

Variable	Observed values			Change from baseline				
	Control group (*n* = 37)	Test group (*n* = 36)	*p* value**	Control group	*p* value*	Test group	*p* value*	*p* value**
Hair luster (L_BNT_)								
At week 0	12.04 ± 3.16	11.67 ± 3.28	0.631^1^					
At week 24	12.57 ± 2.44	13.32 ± 2.79	0.228^1^	0.54 ± 1.87	0.089^3^	1.65 ± 2.30	<0.001^3^	0.027^1^
Hair elasticity (gf/mm^2^)								
At week 0	116.19 ± 26.10	111.33 ± 27.85	0.444^1^					
At week 24	118.14 ± 21.21	121.60 ± 28.92	0.560^1^	1.95 ± 15.45	0.448^3^	10.27 ± 16.40	0.001^3^	0.029^1^
Subjective satisfaction (score)								
At week 0	50.62 ± 13.74	51.34 ± 14.23	0.827^1^					
At week 24	40.16 ± 13.39	33.53 ± 13.12	0.036^1^	−10.45 ± 10.49	<0.001^3^	−17.81 ± 14.35	<0.001^3^	0.015^2^

Data were expressed mean ± standard deviation or frequency (proportion).

Shapiro–Wilk’s test was employed for test of normality assumption.

^*^p values were compared within each group.

^**^p values were compared between groups.

^1independent t-test.^

^2Mann–Whitney U test.^

^3paired t-test.^

### Safety evaluation

**Figure 3:**
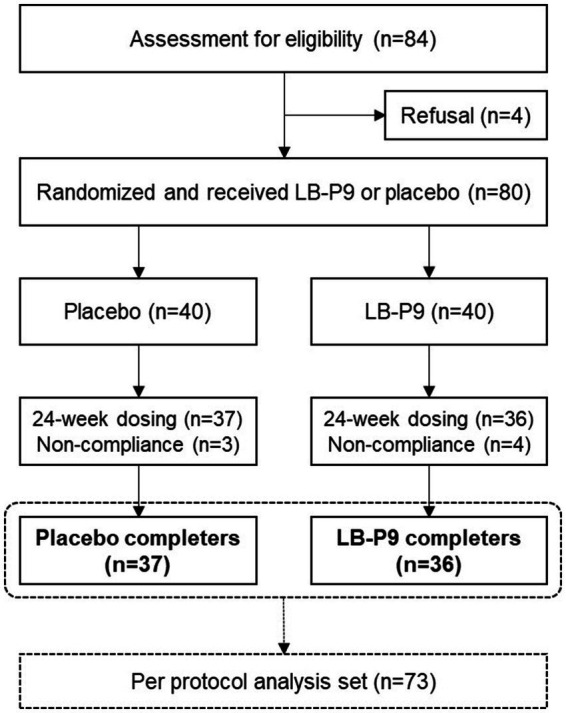
Clinical trial flowchart.

No significant adverse reactions were reported in this clinical trial. Laboratory test results were compared between groups based on changes from baseline, and no significant differences were observed in the changes of evaluation variables between groups. Furthermore, no abnormal findings were observed in the individual results of participants. During the medical history and physical examination of participants, no specific adverse reactions or abnormal cases were observed. No dropouts owing to adverse reactions were reported throughout the study (Figure 3).

Regarding objective signs observed by the investigator, only one participant in the control group exhibited papules at 12 weeks after taking supplements. This was attributed to symptoms of scalp inflammation; however, its relevance to the control supplement was deemed irrelevant. Conversely, some participants reported subjective skin symptoms, including itching, stinging, burning sensation, and roughness at 12 and 24 weeks (Table 3). These subjective symptoms were reported by a few participants and were mild, but they spontaneously improved over time without treatment or intervention.

**Table 3:** Safety assessment of objective signs and subjective symptoms.

Variable			FAS observed value		
			Control group (*n* = 40)	Test group (*n* = 40)	*p-*value**
Objective signs	Erythema	At week 12	0 (0%)	0 (0%)	–
		At week 24	0 (0%)	0 (0%)	–
	Edema	At week 12	0 (0%)	0 (0%)	–
		At week 24	0 (0%)	0 (0%)	–
	Papule	At week 12	1 (2.7%)	0 (0%)	1.000^1^
		At week 24	0 (0%)	0 (0%)	–
	Pustule	At week 12	0 (0%)	0 (0%)	–
		At week 24	0 (0%)	0 (0%)	–
Subjective symptoms	Itching	At week 12	3 (7.7%)	2 (5.3%)	1.000^1^
		At week 24	1 (2.7%)	1 (2.6%)	1.000^1^
	Stinging	At week 12	0 (0%)	0 (0%)	–
		At week 24	0 (0%)	1 (2.6%)	1.000^1^
	Burning	At week 12	0 (0%)	0 (0%)	–
		At week 24	1 (2.7%)	0 (0%)	0.493^1^
	Roughness	At week 12	0 (0%)	1 (2.6%)	0.494^1^
		At week 24	0 (0%)	1 (2.6%)	1.000^1^

Data were expressed mean ± standard deviation or frequency (proportion).

Shapiro–Wilk’s test was employed for test of normality assumption.

^**^p values were compared between groups.

^1Fisher’s exact test.^

## Discussion

Interest in hair loss prevention is growing alongside the desire to maintain healthy hair. Hair is crucial in body image and is affected by numerous physiological and medical factors, scalp care habits, hair product usage, techniques, environmental conditions, and nutritional factors, contributing to hair health. Healthy hair is typically characterized by a shiny and smooth texture, usually with clean-cut ends or tapered tips. Key elements of hair health include texture, structure, and viability, which are associated with hair surface and cortex properties. In a broader sense, the scalp and hair follicle health are crucial to hair health. Hair health is influenced by factors such as aging-related graying and hair loss, which reflect hair density and thickness (1, 2).

Probiotics—beneficial live microorganism strains mainly used to bolster digestive health and overall well-being— may, among others, exert their effect by inhibiting the proliferation of harmful bacteria. Recent reports suggest that certain probiotics can also improve skin health and alleviate inflammatory skin conditions (23–26). Furthermore, recent studies have evaluated the potential of probiotics on hair health, aiming to expand their role as functional food sources. Hair growth is intricately linked to the cycling of anagen, catagen, and telogen phases, and maintaining optimal hair health requires a delicate balance of various factors. This balance involves genetic influences, micro-inflammatory environments affecting the immune system, and oxidative stress systems. Recent findings suggest that probiotics may promote hair growth by modulating the microenvironment of hair follicles (11, 27, 28).

The latest systematic review and meta-analysis examining the efficacy of probiotics for hair health reported improvements in dandruff, scalp serum secretion, and enhancements in hair count, thickness, and density (15). This comprehensive review included four *in vivo* studies and four randomized controlled trials, providing robust evidence of the positive effects of probiotics on hair, particularly regarding hair growth and dandruff alleviation. Probiotic strains examined in these studies include *Lacticaseibacillus paracasei, Leuconostoc holzapfelii, Leuconostoc mesenteroides, Latilactobacillus sakei, Bifidobacterium lactis,Lactobacillus acidophilus, Lacticaseibacillus rhamnosus and Lactiplantibacillus plantarum.* These strains were examined in recent studies assessing the efficacy of probiotics in improving hair growth and alleviating dandruff (14, 20, 29–34).

In this randomized, double-blind, placebo-controlled clinical trial, the probiotics used were *L. curvatus* LB-P9 5 billion CFU/day. No previous clinical trials have specifically addressed hair growth using this strain. The authors have reported findings from *in vitro* tests conducted prior to clinical trials, indicating that LB-P9 increases hair cell proliferation and enhances the expression of VEGF and FGF-7 by hair cells. Furthermore, findings from mouse experiments confirmed that oral administration of *L. curvatus* LB-P9 led to increased hair growth rate, hair follicle count, and dermal layer thickness of the skin while ensuring safety. Molecular mechanisms observed in mouse skin tissue indicated that systemic consumption of LB-P9 stimulates the production of hair growth factors. This includes VEGF, IGF-1, and the antioxidant enzyme superoxide dismutase through activation of the *Wnt*/*β*-catenin pathway (35). A similar report from *in vitro* experiments with a different strain, *L. paracasei*, indicates increased human follicle dermal papilla cell proliferation and secretion of VEGF and IGF-1 (21).

Over the 24-week period, participants consuming LB-P9 at appropriate doses demonstrated statistically significant improvements in hair luster, hair elasticity, and participant satisfaction within the test group compared to the control group. In the subgroup analysis, while the male group and female with long hair groups did not achieve the primary outcome, the female with short hair group showed a significant improvement in luster after 24 weeks. Typically, men tend to have coarser and thicker hair strands, and women have fine hair, which generally means a thinner cuticle layer. Considering that women with fine hair and shorter length might have smaller total amount of cuticle than other groups, it was suggested that the same target dose of probiotics may have led to more significant improvement in this group. It is clear that the small number of participants in each subgroup was a major limitation. Safety evaluations, including adverse event monitoring, clinical laboratory tests, and participant questionnaires, revealed no adverse effects associated with medium-term consumption during the 24-week study period. However, further clinical studies on long-term consumption are deemed necessary. Randomized, placebo-controlled, large-scale trials evaluating efficacy through parameters such as hair count, density, and thickness, alongside hair health, are warranted to determine whether oral supplementation of *L. curvatus* LB-P9 directly affects hair growth.

In conclusion, regular consumption of *L. curvatus* LB-P9 may offer evidence of improving hair health by enhancing hair luster and elasticity, suggesting its potential benefits for overall hair health.

## Data Availability

The original contributions presented in the study are included in the article/Supplementary material, further inquiries can be directed to the corresponding author.
