# Supplementary material for: Efficacy and safety of Latilactobacillus curvatus LB-P9 on hair health: a randomized, double-blind, placebo-controlled clinical trial
Source: Front Nutr. 2024 Nov 12;11:1447863. doi: 10.3389/fnut.2024.1447863 (PMC11600313; doi:10.3389/fnut.2024.1447863)

**Supplement 1.** Hair damage evaluation score based on exposure to risk factors

|  | **1 or more times every 6 weeks** | **Once every 6 weeks to 3 months** | **Less than once every 3 months** |
| --- | --- | --- | --- |
| **Bleaching** | +10 point | +5 point | +1 point |
| **Perming** | +10 point | +5 point | +1 point |
| **Coloring** | +10 point | +5 point | +1 point |
| **Hair cut** | -3 point | -2 point | -1 point |
|  | **5 or more times per week** | **1-5 times per week** | **Less than once per week** |
| **Flat iron** | +5 point | +3 point | +1 point |
| **Blow dry** | +3 point | +2 point | +1 point |
| **Combing/brushing** | +3 point | +2 point | +1 point |
| **Washing** | +3 point | +2 point | +1 point |
| **Deep conditioning treatment** | -5 point | -3 point | -1 point |
| **Rinse off conditioner** | -3 point | -2 point | -1 point |
| **Heat protection spray** | -3 point | -2 point | -1 point |
|  | **≥ 5 h per day** | **1–5 h per day** | **< 1 h per day** |
| **Sun exposure** | +5 point | +3 point | +1 point |

**Supplement 2.** Questionnaire about participant satisfaction score for hair health

| The following questions are aimed at assessing effectiveness through your subjective opinions before and after consuming the test supplements for this study. Please indicate your score for each question by drawing a vertical line. (1 point: Not at all, 10 point: Very much so) |
| --- |
| Q1. Do you believe your hair is damaged? |
| Q2. Is it difficult to comb your hair smoothly, and do you feel resistance when combing it after fully drying it? |
| Q3. Do you feel that your hair lacks shine? |
| Q4. Do you believe your hair is prone to breakage? |
| Q5. Do you think your hair is dry? |
| Q6. Do you feel that your hair lacks elasticity? |
| Q7. Do you feel that the ends of your hair are prone to splitting? |
| Q8. Do you feel that your hair texture is rough? |

**Supplement 3.** Changes in hair elasticity and satisfaction score for hair health according to hair damage score (7 to 18 points) at 24 weeks. [Subject *n*=62, Data were expressed mean ± standard deviation or median (IQR). * *P* values were compared between groups. * *P* <0.05 by t-test and Mann Whitney U test.].


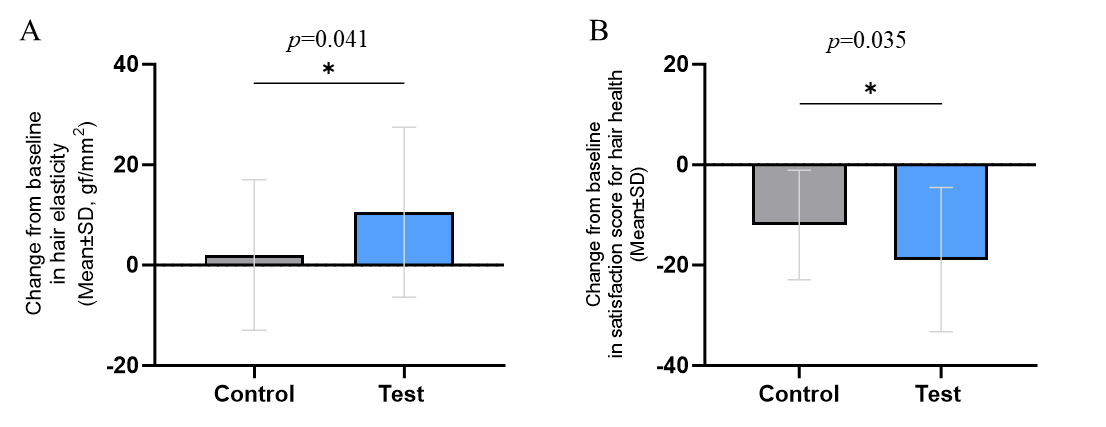

Supplement: Supplementary file 1 [file Data_Sheet_1.docx]
